# Facultative bacterial endosymbionts shape parasitoid food webs in natural host populations: A correlative analysis

**DOI:** 10.1111/1365-2656.12875

**Published:** 2018-07-16

**Authors:** Zhengpei Ye, Ines M. G. Vollhardt, Nadia Parth, Oskar Rubbmark, Michael Traugott

**Affiliations:** ^1^ Mountain Agriculture Research Unit Institute of Ecology University of Innsbruck Innsbruck Austria; ^2^ Agroecology, Department of Crop Sciences Georg‐August‐University Göttingen Göttingen Germany

**Keywords:** aphid, defensive symbiosis, ecological networks, host–parasitoid interactions, hyperparasitoid, parasitoid community

## Abstract

1. Facultative bacterial endosymbionts can protect their aphid hosts from natural enemies such as hymenopteran parasitoids. As such, they have the capability to modulate interactions between aphids, parasitoids and hyperparasitoids. However, the magnitude of these effects in natural aphid populations and their associated parasitoid communities is currently unknown. Moreover, environmental factors such as plant fertilization and landscape complexity are known to affect aphid–parasitoid interactions but it remains unclear how such environmental factors affect the interplay between aphids, parasitoids and endosymbionts.
2. Here, we tested whether facultative endosymbionts confer protection to parasitoids in natural populations of the English grain aphid, *Sitobion avenae*, and if this is affected by plant fertilization and landscape complexity. Furthermore, we examined whether the effects of facultative endosymbionts can cascade up to the hyperparasitoid level and increase primary‐hyperparasitoid food web specialization.
3. Living aphids and mummies were collected in fertilized and unfertilized plots within 13 wheat fields in Central Germany. We assessed the occurrence of primary parasitoid, hyperparasitoid and endosymbiont species in aphids and mummies using a newly established molecular approach.
4. Facultative endosymbiont infection rates were high across fields (~80%), independent of whether aphids were parasitized or unparasitized. Aphid mummies exhibited a significantly lower share of facultative endosymbiont infection (~38%). These findings suggest that facultative endosymbionts do not affect parasitoid oviposition behaviour, but decrease parasitoid survival in the host. Facultative endosymbiont infection rates were lower in mummies collected from fertilized compared to unfertilized plants, indicating that plant fertilization boosts the facultative endosymbiont protective effect. Furthermore, we found strong evidence for species‐specific and negative cascading effects of facultative endosymbionts on primary and hyperparasitoids, respectively. Facultative endosymbionts impacted parasitoid assemblages and increased the specialization of primary‐hyperparasitoid food webs: these effects were independent from and much stronger than other environmental factors.
5. The current findings strongly suggest that facultative endosymbionts act as a driving force in aphid–parasitoid–hyperparasitoid networks: they shape insect community composition at different trophic levels and modulate, directly and indirectly, the interactions between aphids, parasitoids and their environment.

## 1 INTRODUCTION

Heritable facultative bacterial endosymbionts are common in many insect groups (Duron & Hurst, 2013; Zytynska & Weisser, 2016). Unlike obligate symbionts, these are not essential for insect growth or development, but can confer benefits to their hosts such as improved fitness on specific host plants (Tsuchida, Koga, & Fukatsu, 2004), heat tolerance (Montllor, Maxmen, & Purcell, 2002) and resistance to natural enemies (Oliver, Degnan, Burke, & Moran, 2010; Xie, Vilchez, & Mateos, 2010). These endosymbionts can, in addition, also impose a cost to their hosts such as negative effects on longevity (Vorburger & Gouskov, 2011) and reproduction (Simon et al., 2011).

The effect of facultative endosymbionts has been studied for aphid–parasitoid interactions, where these bacteria can kill developing parasitoid eggs or larvae inside their living aphid host (Oliver, Smith, & Russell, 2014; Oliver et al., 2010). To date, four bacterial species have been shown to protect aphids against parasitoids: *Hamiltonella defensa* (Oliver, Degnan, Hunter, & Moran, 2009; Oliver, Russell, Moran, & Hunter, 2003), *Regiella insecticola* (Ferrari, Darby, Daniell, Godfray, & Douglas, 2004; Vorburger, Gehrer, & Rodriguez, 2010), the aphid X‐type symbiont (PAXS; Heyworth & Ferrari, 2015) and *Serratia symbiotica* (Oliver et al., 2003).

The protection conferred by facultative endosymbionts varies between parasitoid species (Oliver et al., 2014): for example, *H. defensa* protects *Aphis craccivora* from *Binodoxys communis* and *Binodoxys koreanus* but not from *Aphidius colemani* and *Lysiphlebus orientalis* (Asplen et al., 2014). Parasitoids can also respond behaviourally to facultative endosymbionts: for example, oviposition in infected aphids can be avoided (Lukasik, Dawid, Ferrari, & Godfray, 2013), multiple eggs can be laid into the same aphid to increase chances of offspring survival (Oliver et al., 2012), or earlier instar aphids with lower numbers of facultative endosymbionts are attacked (Schmid, Sieber, Zimmermann, & Vorburger, 2012). In addition, facultative endosymbionts are not always lethal to parasitoids (Hansen, Vorburger, & Moran, 2012; Oliver et al., 2009) as the parasitoids can overcome the endosymbiotic defence but then often show sublethal effects such as lower emergence rate, delayed development and reduced body size (Nyabuga, Outreman, Simon, Heckel, & Weisser, 2010; Schmid et al., 2012). As this reduces the quality of the primary parasitoid host, facultative endosymbionts may also affect higher trophic levels such as hyperparasitoids (McLean, Hrcek, Parker, & Godfray, 2017; Rothacher, Ferrer‐Suay, & Vorburger, 2016).

Both laboratory studies and cage experiments have shown that facultative endosymbionts can affect parasitoid communities under semi‐natural conditions (Sanders et al., 2016). However, in the open field, aphids are attacked by complex parasitoid communities, which usually include both primary parasitoids and hyperparasitoids (Gariepy & Messing, 2012; Traugott et al., 2008). So far few studies have shown that facultative endosymbionts can affect natural parasitoid communities and this was carried out by transferring laboratory‐reared aphid clones into the field (Hrcek, McLean, & Godfray, 2016; Rothacher et al., 2016). However, this does not take into account that natural aphid populations are exposed to natural selection processes which can affect facultative endosymbiont infection levels and interactions between aphids and higher trophic levels. Therefore, the effects of endosymbionts observed on aphid laboratory clones may not give a complete picture of the functional role of facultative endosymbionts in field populations of aphids and their associated parasitoid networks (Oliver et al., 2014). In recent times, negative correlations of facultative endosymbiont prevalence and parasitism rates in fields were reported for natural pea aphid populations (Smith et al., 2015); however, an assessment of facultative endosymbiont infection and parasitism in individual host aphids is so far missing.

Although laboratory and field studies have elucidated several mechanisms involved in aphid facultative endosymbiont–parasitoid interactions, little is known about the occurrence and the role of facultative endosymbionts in field populations of aphids and their associated parasitoid networks (Oliver et al., 2014). In recent times, we understood environmental factors such as plant diversity can affect aphid facultative endosymbiont communities (Zytynska et al., 2016). For other factors such as plant fertilization and landscape complexity which are known to affect aphids, parasitoids and the interactions between them (e.g. Aqueel & Leather, 2011; Aqueel et al., 2015; Lohaus, Vidal, & Thies, 2013), we still have little knowledge on how these factors might impact the facultative endosymbionts in natural aphid populations. Moreover, the community‐level consequences of facultative endosymbionts on aphid–primary parasitoid–hyperparasitoid networks in natural aphid populations are poorly understood as well as how important these are in comparison with factors such as landscape complexity and plant fertilization (Butler, Garratt, & Leather, 2012; Tscharntke et al., 2012).

Here, we address these knowledge gaps for field populations of one of the most important pests in cereal crops, the English grain aphid *Sitobion avenae* (Poehling, Freier, & Klüken, 2007). Although facultative endosymbionts have been shown to protect a variety of aphid species against different parasitoid species (Oliver et al., 2014), so far in *S. avenae*, the tested four strains of *H. defensa* conferred no physiological resistance against species of parasitoids in laboratory experiments (Lukasik et al., 2013). However, a possible defensive function of facultative endosymbionts can still be expected in natural *S. avenae* populations, as a much wider spectrum of parasitoid species attacks cereal aphids providing the potential for species‐specific protective effects in the field compared to laboratory experiments (Oliver et al., 2014). On the other hand, *S. symbiotica* has not been included in our assay, as *S. symbiotica* has not been reported in *S.avenae* from an extensive survey (Henry, Maiden, Ferrari, & Godfray, 2015). Also, this endosymbiont has been considered to be more important to provide other benefits to its aphid hosts, as there is a lack of strong protective phenotypes and limited defensive factors in its genome (Oliver et al., 2014).

In this study, we collected living and mummified *S. avenae* from winter wheat fields situated in complex and simple landscapes. In addition, fertilized or unfertilized experimental treatments were established within each field. All aphids and mummies collected were screened for three species of facultative endosymbionts and a range of primary and hyperparasitoid species using a newly developed molecular approach which was sensitive enough to allow reliable detection of a single parasitoid egg and facultative endosymbiont DNA in both living and mummified aphids (Ye et al., 2017). Using this experimental set‐up, we tested three hypotheses:

H1) Facultative endosymbionts will confer protection against parasitoids in *S. avenae*. Therefore, our prediction is that facultative endosymbiont infection rates in mummies will be lower compared to that of living parasitized aphids and that of living parasitized aphids will be lower compared to living unparasitized aphids.

H2) Facultative endosymbiont infection rates will be higher in aphids collected from fertilized compared with unfertilized plots, and higher in complex compared to simple landscapes. This is expected because plant fertilization has often been shown to increase aphid performance (Aqueel & Leather, 2011), which should make facultative endosymbionts more “affordable” for aphids. Furthermore, aphids living in cereal fields situated in complex landscapes often have higher parasitism rates compared to simple landscapes (e.g. Plecas et al., 2014), and this higher parasitism pressure should increase the endosymbiont infection rate as indicated by laboratory experiments (Oliver, Campos, Moran, & Hunter, 2008).

H3) We expect lower hyperparasitism rates in parasitized aphids/mummies containing facultative endosymbionts in comparison with uninfected ones and an increased specialization of primary‐hyperparasitoid food webs when facultative endosymbiont infection rates are high. Compared to uninfected aphids, the hyperparasitoids are more likely to avoid endosymbiont‐infected aphids because of the lower quality of its hosts, primary parasitoids (Harvey, Gols, Snaas, Malcicka, & Visser, 2015; Otto & Mackauer, 1998), which can be sublethally affected by facultative endosymbionts. Moreover, the reduction in the quality of the primary parasitoid host should intensify the competition between hyperparasitoid resulting in a higher specialization of primary‐hyperparasitoid food webs.

## 2 MATERIALS AND METHODS

### 2.1 Study design

A field experiment was conducted in 13 winter wheat fields situated in seven complex and six simple landscapes, in Lower Saxony, Germany, in 2013. Landscape complexity was calculated as the percentage of semi‐natural (e.g. field margins, hedges, pastures and flowering strips) land within a radius of 500 m around each field using arcview (Version 10.0; ESRI Geoinformatik GmbH, Hannover, Germany). Complex landscapes contained more semi‐natural landscape elements which ranged between 25% and 44% around each field while this was 2%–19% for simple landscapes (Supporting Information Table S1). Complex and simple landscape fields contain 43%–72% and 69%–92% arable land, respectively, as well as 3%–18% and 3%–14% other landscape structures such as settlements, streets and rivers. The fields were located within an area of 375 km^2^ around Göttingen with a similar climate. In each field, an experimental area of 50 × 100 m was left free of herbicides, insecticides and fertilizers. Within each experimental area, eight plots of 6 × 6 m each were established in two adjacent rows (distance between plots at least 8 m [range: 8–10 m], distance between plots and edges of field at least 7 m [range: 7–19 m]). Within each field, four of the plots were fertilized by hand with “Blaukorn” (Compo GmbH, Münster, Germany), a complex fertilizer comprised of nitrogen, phosphorus and potassium. Fertilized plots were alternated with unfertilized plots except in three fields where some plots of the same treatments had to be located together to avoid effects of increased soil humidity or slopes. Fertilization was administered according to recommended agricultural practices for winter wheat and took place between 2 and 15 April 2013 (1.8 kg N/plot) and between 8 and 10 May 2013 (3 kg N/plot).

Aphids and mummies were collected for molecular analysis in all plots at wheat milk ripening (02 July–06 July 2013) and dough ripening (08 July–13 July 2013) stage. Randomly selected wheat ears with aphid colonies were cut and individually stored in 50 ml tubes at −20°C. Aphids were picked equally from each tube and stored individually in 96‐well plates at −80°C for molecular analysis. Only higher instar aphids (3rd or 4th instar nymphs and adults) were selected to obtain a balanced set of aphids per plot. Sampling continued until at least 20–50 aphids/plot were found. Mummies were collected from 120 randomly picked tillers in each plot. As the mummification rate was quite low, sampling of mummies was continued until at least 5–15 mummies/plot were found. In the field “C4,” there were no aphids at the second sampling date, therefore the sampling was skipped for this date (Supporting Information Table S1).

### 2.2 Molecular analysis

The molecular detection and identification of primary parasitoids, hyperparasitoids and facultative endosymbionts within field‐collected aphids and mummies followed the protocols described in Ye et al. (2017). In short, all aphids and mummies were DNA extracted using a Chelex‐based extraction protocol and the DNA extracts were subjected to a modified two‐step multiplex PCR (MP‐PCR) system. The primers targeting aphids, *Metopolophium dirhodum* and *Rhopalosiphum padi,* were removed from the MP‐PCR assays compared to the original protocols (Ye et al., 2017), as only *S. avenae* was present in the investigated fields. In addition, as the original protocol in Ye et al. (2017) has been shown to allow a reliable detection of facultative endosymbionts in mummified *Acyrthosiphon pisum*, which is slightly larger than *S. avenae*, a further test to confirm the reliability of facultative endosymbiont detection in mummies of a different, smaller, aphid species was conducted (details see Supporting Information). Within every batch of 96 samples, at least two extraction negative controls, target DNA (PCR positive control) and molecular grade water (PCR negative control), were included. In addition, at least two (range 2–34 per taxon; Supporting Information Table S2) randomly selected PCR products per target were sequenced to confirm the taxon identity. As only one PCR product for *Coruna clavata* was obtained, there was only one amplicon which could be sequenced (Supporting Information Table S2). All amplicons sequenced turned out to have the correct identity.

### 2.3 Data analysis

All data analysis was conducted in r version 3.2.0 (R Core Team 2015). To assess the parasitoid oviposition, primary and hyperparasitism were defined as the molecular detection of primary parasitoids in living aphids and the detection of hyperparasitoids in primary parasitized aphids/mummies, respectively. Generalized linear mixed‐effects models (“glmer”) with a binomial probability distribution were used for regression‐based analysis (Zuur, Ieno, Walker, Saveliev, & Smith, 2009). To analyse the response of endosymbiont infection in different aphid parasitism types (living unparasitized aphids, living parasitized aphids and mummies), fields/plots/tubes were used as random factors to avoid nonindependence of endosymbiont infection in samples from a same aphid colony. Regarding analysing the effects on primary and hyperparasitism rates in endosymbiont infection types (uninfected, *H. defensa*‐infected, and *R. insecticola*‐infected), fields/plots were used as random factor. Landscape complexity, plant fertilization and sampling date were considered as three abiotic environmental fixed factors in all models. Models were fitted for all possible factor combinations as well as the null assumption. From these, the best fitting model was selected based on Akaike information criterion (AIC). Model fitting was conducted using the r package “lme4” (Bates, Machler, Bolker, & Walker, 2015). The model assumptions were checked according to Zuur, Ieno, and Elphick (2010) using binned residual plots (r package “arm”; Gelman & Su, 2016). To test for differences between groups post hoc tests (Tukey) were conducted and corrected for false discovery rate using package “multcomp” (Hothorn, Bretz, & Westfall, 2008).

The species composition of both primary and hyperparasitoids was assessed using distance‐based redundancy analysis (dbRDA) ordinations based on Bray–Curtis dissimilarities using the package “vegan” (Oksanen et al., 2016). The contribution of each species to the structural differences that exists between two parasitoid communities in different facultative infection status (beta diversity) was assessed using similarity percentages (“simper”) in the r package “vegan” (Oksanen et al., 2016). The “simper” analysis is also based on Bray–Curtis dissimilarities and lists the average abundance of each species in the community and its contribution to the total dissimilarity (cumulative contribution). At last, this analysis provides the permuted *p* value, showing how significant the cumulative contribution of each species between the two communities is. The significance of all terms in dbRDA and the significance of the contribution of each parasitoid species in “simper” were tested using 1,000 permutations. In dbRDA, facultative endosymbiont infection type (uninfected, *H. defensa*‐infected, *R. insecticola*‐infected), field identity, sampling date, plant fertilization and landscape complexity were used as explanatory variables. Samples infected with both *H. defensa* and *R. insecticola* were not considered in the analyses due to a very low rate of superinfection (see results section), except when analysing the effect on facultative endosymbionts in general. In the parasitoid assemblage analyses, only parasitoid species that were observed more than five times were included, and plots were only included if more than three individual aphids had been molecularly analysed.

Primary‐hyperparasitoid bipartite food webs were assessed using a resampling approach: as primary and hyperparasitism rates are often low in cereal aphid populations (typically below 25%; e.g. Traugott et al., 2008; Derocles et al., 2014), comparison between food web parameters of fertilized and unfertilized plots situated in simple and complex landscapes was difficult using standard statistics. As food web metrics can be affected by sample size (Tylianakis, Laliberte, Nielsen, & Bascompte, 2010) and as food webs from individual fields in our dataset differed greatly in size, food web metrics were calculated from 200,000 random draws with replacement of 100 individual interactions, resulting in a total of 200,000 resampled food webs. All the explanatory variables in the original dataset, including endosymbiont infection, fertilization, landscape complexity and sampling date, were implemented as binomial variables. Therefore, each resampled food web was assembled from interactions drawn from the original dataset, such that each drawn individual retained its explanatory variables in a separate matrix to allow the proportional assembly for binomial variables to be calculated for each resampled food web. These proportional assembled explanatory variables represent the level of endosymbiont infection, fertilization, landscape complexity and sampling time for each resampled food web. In addition, network level specialization (*H*_*2*_ *′*; Blüthgen, Menzel, & Blüthgen, 2006) was calculated for each resampled food web using the R package “bipartite” (Dormann, Gruber, & Fründ, 2008). Afterwards, linear models (“lm”) were used for testing the effects of the explanatory variables, their interactions on *H*_*2*_ *′* and the model simplification was conducted as described above. The advantage of this approach is that it allows for testing the correlation between changes in explanatory variables and emergent food web properties while retaining a similar sample size across food webs and reducing the effects of otherwise problematic random (between‐site) differences.

## 3 RESULTS

**Figure 1:**
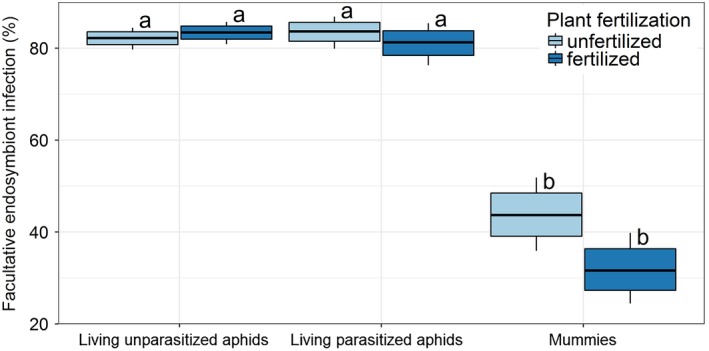
Endosymbiont infection rates in living unparasitized aphids, living parasitized aphids and mummies collected within unfertilized (light blue) and fertilized plots (dark blue). Different letters indicate significant differences between groups (*p* < 0.05); note, however, that the difference between the infection rates in mummies from unfertilized and fertilized plants was almost significant at *p* = 0.06. The bottom/top of the box and the whiskers correspond to the 75% confidence interval and 95% confidence interval, respectively

The infection of 6,433 living *S. avenae* and 563 mummies collected from 13 winter wheat fields with facultative endosymbionts was assessed. Overall, 40.1% and 39.5% of the living aphids and 29.8% and 7.64% of the mummies tested positive for *H. defensa* and *R. insecticola*, respectively. DNA of both *H. defensa* and *R. insecticola* (“superinfection”) was detected in 0.89% of living aphids and 0.53% of mummies while no DNA of PAXS could be amplified from any of the samples tested. The facultative endosymbiont detection rate was similar in living unparasitized and parasitized aphids, whereas the percentage of facultative endosymbiont‐positive individuals dropped by ~50% in mummies compared to living unparasitized (*df*_5(used),6987(residual)_, *z* = −10.21, *p* < 0.001) and parasitized aphids (*z* = −9.36, *p* < 0.001; Figure 1; details of the model fitting see Supporting Information Table S3). In mummies, facultative endosymbiont infection was almost significantly lower in fertilized (mean 31.6%, CI_0.05/0.95_ 24.5/39.8%) compared to unfertilized plots (mean 43.7%, CI_0.05/0.95_ 35.9/51.8%, *z* = −2.10, *p* = 0.060; Figure 1; details of the model fitting see Supporting Information Table S3). Landscape complexity and sampling date did not explain sufficient variation in facultative endosymbiont detection rates according to the model simplification and were therefore omitted from the final models.

**Figure 2:**
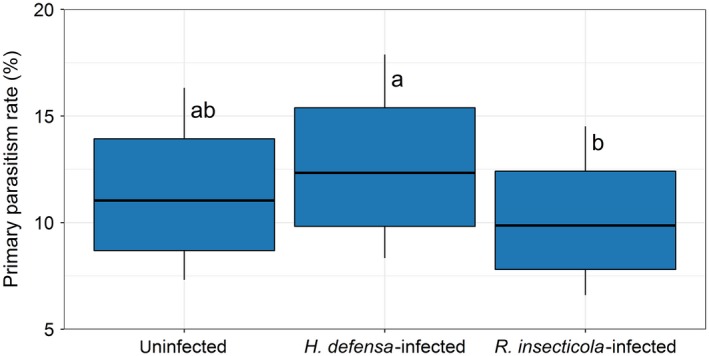
Primary parasitism rate within uninfected, *Hamiltonella defensa*‐infected and *Regiella insecticola*‐infected *Sitobion aveane* living aphid samples. Different letters indicate significant differences between groups (*p* < 0.05). The bottom/top of the box and the whiskers correspond to the 75% confidence interval and 95% confidence interval, respectively

**Figure 3:**
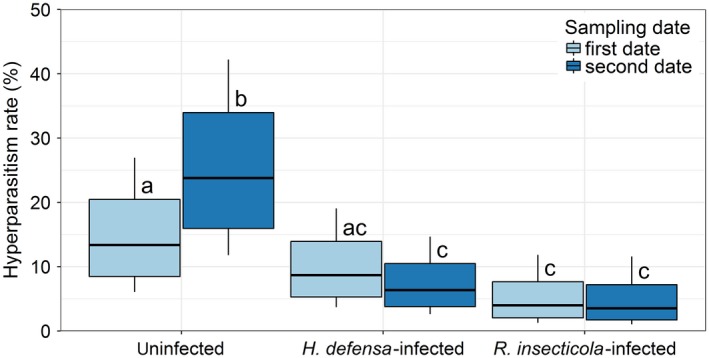
Hyperparasitism rate within uninfected, *Hamiltonella defensa*‐infected and *Regiella insecticola*‐infected *Sitobion aveane* aphid/mummy samples collected at the wheat milk ripening stage (the first sampling date; light blue) and dough ripening stage (the second sampling date; dark blue). Different letters indicate significant differences between groups (*p* < 0.05). The bottom/top of the box and the whiskers correspond to the 75% confidence interval and 95% confidence interval, respectively

For analysing the effects of endosymbiont and environment factors on primary parasitism rates, 6,319 living aphids were used: the field “C4,” situated in a complex landscape, was excluded from the analyses, as no parasitized aphids could be found (Supporting Information Table S1). Primary parasitism rate did not differ between uninfected aphids and aphids infected with either, or both, species of facultative endosymbionts; however, the primary parasitism rate of *H. defensa*‐infected aphids was higher compared to *R. insecticola*‐infected aphids (*df*_4,6312_, *z* = −3.05, *p* = 0.007; Figure 2; details of the model fitting see Supporting Information Table S3). Landscape complexity did not explain sufficient variation in primary parasitism rates according to the model simplification, and therefore, it was not included in the final models. In total, 3% of the aphid and mummy samples (*n* = 206) were tested positive for hyperparasitoids. The effects of endosymbiont and environment factors on hyperparasitism rates were analysed using 1,312 primary parasitized samples. The effect of facultative endosymbionts on hyperparasitism rate differed between sampling dates: no significant difference was found for hyperparasitism rates between uninfected and *H. defensa*‐infected samples, whereas hyperparasitism rates are higher in uninfected compared to *R. insecticola*‐infected (*df*_5,1304_, *z* = −2.78, *p* = 0.015) samples at the first sampling date. At the second sampling date, the hyperparasitism rates in uninfected samples were significantly higher compared to *H. defensa*‐ (*df*_5,1304_, *z* = −5.15, *p* < 0.001) and *R. insecticola*‐infected (*z* = −4.00, *p* < 0.001) samples collected (Figure 3; details of the model fitting see Supporting Information Table S3). Landscape complexity and plant fertilization did not explain sufficient variation in hyperparasitism rates according to the model simplification. Thus, these variables were not included in the final models.

**Figure 4:**
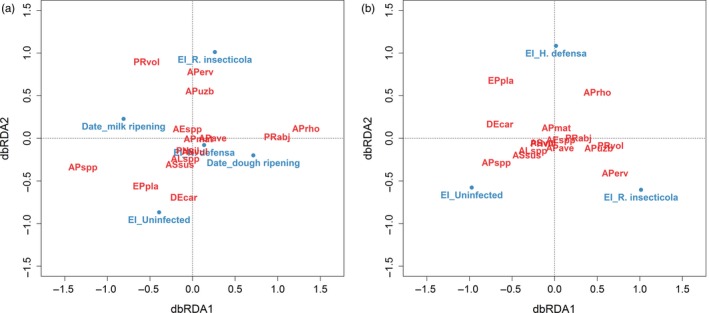
(a) Partial distance‐based redundancy analysis (dbRDA) ordinations based on Bray–Curtis dissimilarities showing correlations of parasitoid species (red) composition with environmental variables, including sampling date (Date) and endosymbiont infection (EI; plant fertilization and landscape complexity were partitioned; *p* = 0.001). (b) Partial dbRDA ordinations showing correlations of parasitoid species (red) composition with endosymbiont infection (blue; sampling date, plant fertilization and landscape complexity were partitioned; *p* = 0.001). Species abbreviations—primary parasitoids: *Aphelinus* spp. (AEspp), *Aphidius avenae* (APave), *Aphidius ervi* (APerv), *Aphidius matricariae* (APmat), *Aphidius rhopalosiphi* (APrho), *Aphidius uzbekistanicus* (APuzb), *Aphidius* spp. (APspp), *Ephedrus plagiator* (EPpla), *Praon abjectum* (PRabj) and *Praon volucre* (PRvol); hyperparasitoids: *Alloxysta *spp. (ALspp), *Asaphes vulgaris* (ASvul), *Asaphes suspensus* (ASsus), *Dendrocerus carpenteri* (DEcar) and *Phaenoglyphis villosa* (PHvil)

When assessing the effect of different environmental variables on parasitoid assemblages in dbRDA, field identity, sampling date, plant fertilization, landscape complexity and facultative endosymbiont infection together explained 10.4% of the variance found in the parasitoid communities (*df*_15,398_, *F* = 3.09, *p* = 0.001). Excluding field identity, all other variables cumulatively explained 4.85% of the variance (*df*_5,408_, *F* = 4.16, *p* = 0.001), in which sampling date, plant fertilization and landscape complexity dominated the first dbRDA axis (explained variation 2.04%), whereas the occurrence of the facultative endosymbionts dominated the second dbRDA axis (explained variation 1.09%). When the effects of landscape complexity and plant fertilization were partitioned out, sampling date and facultative endosymbiont infection explained 3.39% of the variance (*df*_3,408_, *F* = 4.84, *p* = 0.001). *Regiella insecticola* infection did differ between parasitoid species, whereas the effect of *H. defensa* was not strongly correlated with any parasitoid species (Figure 4a). The pure facultative endosymbiont infection effect, after removing sampling date from the analysis, explained 1.56% of the variance found within the parasitoid composition (*df*_2,408_, *F* = 3.34, *p* = 0.001) with the first dbRDA axis discriminating between uninfected and *R. insecticola*‐infected hosts. *Aphidius* spp. and *Dendrocerus carpenteri* had a higher occurrence in uninfected samples while for *Aphidius ervi,* occurrence was more common in *R. insecticola*‐infected aphids. *Ephedrus plagiator* and *A. rhopalosiphi* tended to occur in *H. defensa*‐infected aphids (Figure 4b).

The “simper” analysis showed that eight parasitoid species contributed most to the dissimilarities and their abundances were different between facultative endosymbiont‐infected and uninfected hosts (Table 1): the average abundance of *E. plagiator* was higher in *H. defensa*‐infected samples compared with uninfected ones (1,000 permutations, *p* = 0.001), whereas the occurrence of the hyperparasitoid *D. carpenteri* was lower in these samples (*p* = 0.001). In *R. insecticola*‐infected samples, *Aphidius* spp. was more common than in uninfected samples (*p* = 0.003). The average abundances of *Aphidius uzbekistanicus* (*p* = 0.007), *Praon volucre* (*p* = 0.005) and *Aphidius rhopalosiphi* (*p* = 0.004) were higher in aphids which contained *H. defensa* compared to hosts infected by *R. insecticola*, whereas the opposite was true for *Praon abjectum* (*p* = 0.041).

**Table 1:** Parasitoid species that contributed most to dissimilarities of the parasitoid assemblages in uninfected, *Hamiltonella defensa*‐infected and *Regiella insecticola*‐infected samples. Species with significant effects are highlighted in bold

Group A	Group B	Species	Average abundances in group A	Average abundances in group B	Cumulative contributions	*p*‐Value
Uninfected	*H. defensa*	*Aphidius uzbekistanicus*	0.75	1.11	0.20	0.93
		*Praon volucre*	0.52	0.81	0.35	0.99
		***Ephedrus plagiator***	**0.53**	**0.63**	**0.49**	**0.001**
		*Aphidius* spp.	0.61	0.34	0.62	0.06
		*Aphidius ervi*	0.42	0.40	0.73	0.97
		*Aphidius rhopalosiphi*	0.20	0.34	0.81	0.80
		***Dendrocerus carpenteri***	**0.44**	**0.19**	**0.88**	**0.001**
		*Praon abjectum*	0.17	0.29	0.94	0.95
Uninfected	*R. insecticola*	*A. uzbekistanicus*	0.75	0.82	0.20	0.91
		*P. volucre*	0.52	0.70	0.37	0.66
		***Aphidius*** **spp.**	**0.61**	**0.27**	**0.50**	**0.003**
		*A. ervi*	0.42	0.43	0.62	0.28
		*E. plagiator*	0.53	0.20	0.73	0.96
		*A. rhopalosiphi*	0.20	0.30	0.81	0.93
		*P. abjectum*	0.17	0.32	0.88	0.49
		*D. carpenteri*	0.44	0.05	0.94	0.13
*H. defensa*	*R. insecticola*	***A. uzbekistanicus***	**1.11**	**0.82**	**0.22**	**0.007**
		***P. volucre***	**0.81**	**0.70**	**0.41**	**0.005**
		*A. ervi*	0.40	0.43	0.54	0.10
		*E. plagiator*	0.63	0.20	0.65	0.65
		*Aphidius* spp.	0.34	0.27	0.76	1
		***A. rhopalosiphi***	**0.34**	**0.30**	**0.86**	**0.004**
		***P. abjectum***	**0.29**	**0.32**	**0.94**	**0.041**
		*D. carpenteri*	0.19	0.05	0.97	1

**Figure 5:**
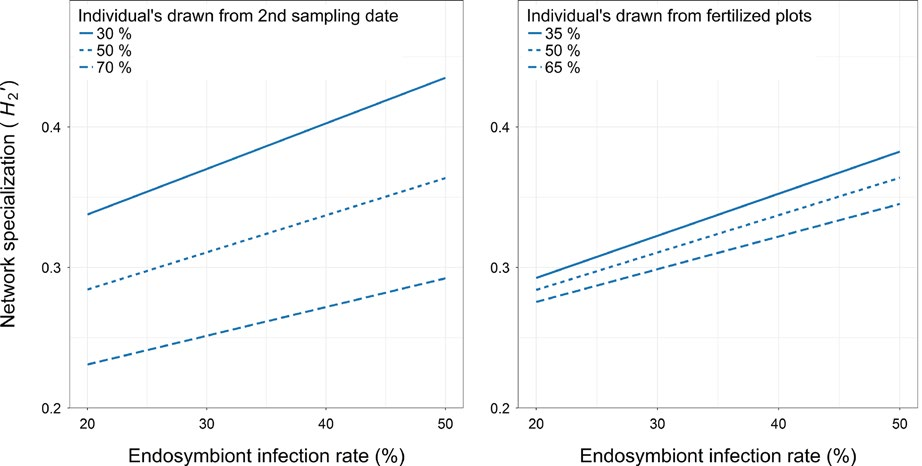
Effects of facultative endosymbiont infection rate in hyperparasitized aphids/mummies based on the sampling date (left panel) and plant fertilization (right panel) on network level specialization index (*H*_*2*_ *′*). Sampling was conducted at two time points: 1st sampling date (wheat milk ripening stage, 02 July–06 July 2013) and 2nd sampling date (wheat dough ripening stage, 08 July–13 July 2013)

The specialization of primary–hyperparasitoid food webs increased with increasing facultative endosymbiont infection rate (*R* ^2^ = 0.34, Pearson correlation = 0.17; Figure 5). This effect became weaker over time and also weaker with plant fertilization (*R* ^2^ = 0.0004; Figure 5). As the analysis was a resampling‐based approach, only the effect sizes and directions are shown.

## 4 DISCUSSION

This study provides the first comprehensive assessment on the occurrence of three facultative endosymbionts and their effects on the assemblages of primary parasitoids and hyperparasitoids in natural *S. avenae* populations. Moreover, we examined the putative effects of landscape complexity and plant fertilization, on the occurrences of facultative endosymbionts and their interaction with parasitoids.

In our first hypothesis, we predicted that *H. defensa* and *R. insecticola* confer protection to parasitoids. We found that overall facultative endosymbiont infection rates were around 80% in living unparasitized and parasitized aphids, while they were approximately half in mummified aphids. From this, we would suggest that either *R. insecticola*,* H. defensa* or both protect aphids from parasitoids under field conditions, even if aphids are killed by parasitoids species of which some might be able to overcome this protection. The protection is, however, not 100% effective, as still approximately half of the facultative endosymbiont‐infected aphids were mummified and killed by parasitoids.

In cases where protection is successful, the immature parasitoid is killed in the host aphid (Hansen et al., 2012; Oliver et al., 2009), and it is thus likely that a selection for the ability to avoid facultative endosymbionts should be occurring among parasitoids (Lukasik et al., 2013). In fact, there are reports that parasitoids avoiding oviposition into *S. avenae* infected by *H. defensa*, even though this facultative endosymbiont does not affect parasitoid development (Lukasik et al., 2013). However, as facultative endosymbiont infection rates were similar between living unparasitized and parasitized aphids, we could not see direct evidence from our study for such avoidance. This might be explained by the parasitoid response to facultative endosymbiont protection varying between species (Asplen et al., 2014; McLean & Godfray, 2015), including either resistance to toxins, behavioural adaptation or both. Thus, in natural parasitoid communities, such effects may be difficult to discern. Furthermore, the facultative endosymbiont infection rates were relatively high (~80%) and aphid densities were low in the experimental fields. This might have caused a trade‐off for parasitoids between the costs of taking the risk of oviposition in infected hosts (Oliver et al., 2003) and searching for uninfected aphids (Lukasik et al., 2013). Even if the parasitoids may be able to detect the facultative endosymbionts, the high facultative endosymbiont infection rate combined with low aphid density may have pushed primary parasitoids to use all hosts available or use other oviposition strategies, such as laying multiple eggs into the same aphids (Oliver et al., 2012) or laying eggs in to earlier instar aphids (Schmid et al., 2012). Note, however, that the availability of alternative hosts such as aphids on weeds in the fields could relax the pressure of parasitoids in finding facultative endosymbiont‐free hosts.

The relationship between facultative endosymbiont prevalence and parasitism also needs to be interpreted cautiously, as the present study did not account for aphid genotype. For example, some pea aphid genotypes have been shown to resist *A. ervi* in the absence of endosymbionts, although the symbiont‐encoded resistance has still been suggested to be the most important mechanism for aphid–parasitoid defence (Martinez, Ritter, Doremus, Russell, & Oliver, 2014). In *S. avenae*, specific host genotype–endosymbiont associations have been found, however, parasitism rate was unaffected by these host genotype–endosymbiont associations (Zepeda‐Paulo, Villegas, & Lavandero, 2017). Bearing this in mind, our results seem not to be affected by endosymbiont–host genotype associations and they indicate that *H. defensa* and *R. insecticola* primarily affect immature parasitoids by killing them within their aphid hosts rather than by avoiding the parasitoids’ oviposition into infected aphids. In addition, molecular methods may overestimate the real biocontrol efficiency of parasitoids on their aphid hosts, as they do not indicate whether parasitoid eggs and larvae survive within the host or if they are killed by host defence mechanisms, which has been discussed earlier (Agusti et al., 2005; Gariepy, Kuhlmann, Gillott, & Erlandson, 2007; Traugott, Kamenova, Ruess, Seeber, & Plantegenest, 2013).

Our results also suggest the effects of *R. insecticola* and *H. defensa* on parasitoids were different. Several previous studies have shown that the strength of protection can differ among facultative endosymbiont species or even between different strains of the same facultative endosymbiont species (reviewed by Oliver et al., 2014). In our study system, *R. insecticola* seems to induce stronger effects on the parasitoid assemblage. It has been shown that the protective function of *H. defensa* depends on the presence of specific bacteriophages called APSEs (*A. pisum* secondary endosymbiont; Degnan & Moran, 2008; Oliver et al., 2009). Here, the samples were not tested for the identities of these bacteriophages, which may have inflated the variability in the protective strengths of facultative endosymbionts. The effects of facultative endosymbionts, including endosymbiont strain information and APSEs identities, in natural aphid and parasitoid populations should be examined in future studies.

The low superinfection rate of the two endosymbiont species detected in the present study does not allow conducting an assessment of its effects on parasitoids. These low rates might also be due to the multiplex PCR system employed as low numbers of endosymbionts might go undetected, for example, in freshly infected aphids. Still, the detection of the dominant endosymbionts was certainly possible and as the protective effect of facultative endosymbionts against parasitoids is correlated to the bacterial number (Schmid et al., 2012), the functionally relevant endosymbionts were detected in the field‐collected aphids.

In our second hypothesis, we tested if facultative endosymbiont infection is positively affected by plant fertilization and landscape complexity. Overall, we could find no support for the latter. However, while only marginally nonsignificant (*p* = 0.060), the facultative endosymbiont infection rates of mummies were lower in fertilized compared to unfertilized plots. This could, for example, be an indication that facultative endosymbionts are more protective for aphids developing on fertilized plants. Thus, plant fertilization may aid aphids in affording facultative endosymbionts and thereby to obtain stronger parasitoid resistance. This may be due to the fact that facultative endosymbionts lack pathways for the production of several amino acids (Degnan, Yu, Sisneros, Wing, & Moran, 2009; Degnan et al., 2010), which they need to take up from the haemolymph of the aphids, and these amino acids are likely to be more available in aphids developing on fertilized plants. In addition, as the facultative endosymbiont numbers within a host can be positively related to the level of protection in aphids (Schmid et al., 2012), and the fitness of aphids is likely to increase with host plant fertilization level (Aqueel & Leather, 2011), this should lead to higher facultative endosymbiont numbers within the host and provide better protection against parasitoids for aphids developing on fertilized plants.

Our third hypothesis, that hyperparasitism rates of parasitized aphids/mummies containing facultative endosymbionts should be lower compared to uninfected samples, was supported as hyperparasitism rate was over 40% greater in the latter (~19%) than in the former (~11% in *H. defensa*‐ and *R. insecticola*‐infected samples together). This suggests a cascading effect of facultative endosymbionts to higher trophic levels and our findings corroborate Rothacher et al. (2016) who also found a lower abundance of hyperparasitoids in *H. defensa*‐infected *Aphis fabae* placed out in the field. It could be that facultative endosymbionts directly kill the hyperparasitoid larvae. In present study, however, it is more likely that these were indirectly affected by the two facultative endosymbiont species: the dominant hyperparasitoids were mummy hyperparasitoids (*mainly D. carpenteri*), which only attack the primary parasitoids inside the mummified aphid (Müller, Adriaanse, Belshaw, & Godfray, 1999) where only the primary parasitoid larvae and hardly any facultative endosymbionts would be still active. Primary parasitoids that survive in facultative endosymbiont‐infected aphids would have less mass, smaller size and slower development compared to conspecifics developing in uninfected aphids (Nyabuga et al., 2010; Schmid et al., 2012). The former are likely to be poor hosts for hyperparasitoids and consequently being avoided, as indicated by our findings. Further experimental work is, however, needed to elucidate the mechanisms for the hyperparasitoid avoidance of primary parasitoids in facultative endosymbiont‐infected aphids proposed here.

With regard to our third hypothesis that increasing endosymbiont infection levels should boost the specialization of primary–hyperparasitoid food webs, it seems that aside from sampling date (*R* ^2^: 0.35), facultative endosymbiont infection (*R* ^2^: 0.34) had a much stronger effect on networks than either plant fertilization (*R* ^2^: 0.03) or landscape complexity (*R* ^2^: 0.02). Here, we argue that these results might indicate a trade‐off for the hyperparasitoids between the cost of searching for a high‐quality host and the benefits procured in finding their host, that is if facultative endosymbiont infection rate is high, hyperparasitoids might attain substantial benefit from finding an optimal host species which, as seen from results, would lead to a higher degree of network specialization. If, on the other hand, facultative endosymbiont infection is low, the benefit of finding an optimal host species might be less which would lead to a lower degree of specialization as the costs of searching for this optimal host species is likely to be proportionally higher.

In our analysis, facultative endosymbiont infection had a significant effect on parasitoid assemblages, although, while stronger than for other environmental variables, the effect size was minor. Other environmental variables such as plant fertilization and landscape complexity may (Aqueel et al., 2015; Plecas et al., 2014) or may not (Lohaus et al., 2013; Rand, van Veen, & Tscharntke, 2012; Vollhardt, Tscharntke, Wäckers, Bianchi, & Thies, 2008) affect parasitoid community structure. The present findings, where approximately one‐third of the explained variability (except field identity) in parasitoid assemblages was explained by facultative endosymbiont infection, do, however, suggest that bacteria can influence parasitoid community assembly. The effect of facultative endosymbiont infection on parasitoid communities did in this case not correlate with sampling date, plant fertilization or landscape complexity, which suggests that facultative endosymbionts affect parasitoid species independently of these other environmental variables such as fertilization or landscape complexity. Previous studies have also shown that facultative endosymbionts can affect aphid–parasitoid communities. For example, a cage‐base experiment suggests that facultative endosymbionts play an important role in the stability of aphid–parasitoid communities (Sanders et al., 2016). Also, Hrcek et al. (2016) and Rothacher et al. (2016) have shown that facultative endosymbionts can affect natural parasitoid communities on the genus and species level, respectively, by transferring laboratory‐manipulated facultative endosymbiont‐infected *A. fabae* or *A. pisum* to natural environments. Besides the species‐specific endosymbiont–parasitoid associations, previous studies have demonstrated facultative endosymbiont–parasitoid genotype‐specific interactions (Rouchet & Vorburger, 2012; Vorburger, Sandrock, Gouskov, Castaneda, & Ferrari, 2009) and that parasitoids adapt rapidly to selection of facultative endosymbionts (Dion, Zele, Simon, & Outreman, 2011; Rouchet & Vorburger, 2014). The effects of facultative endosymbionts on natural parasitoid communities, including parasitoid genotype information, are thus suggested as a next step in future studies.

We also found support for species‐specific facultative endosymbiont–parasitoid effects. For example, *E. plagiator* tolerated *H. defensa* to a greater extent than other parasitoids, and the same seems to be true for *A. rhopalosiphi* and *A. ervi* for *R. insecticola*. *Aphidius* spp. and *D. carpenteri* were putatively the most sensitive to facultative endosymbionts, as they were strongly correlated to uninfected aphids and mummies, respectively. Our study identifies parasitoid species which might differ in specific traits regarding their susceptibility to certain facultative endosymbiont species. For example, the fecundity of the facultative endosymbiont‐“tolerant” parasitoids *E. plagiator* (~160–250 eggs) and *A. rhopalosiphi* (~200 eggs) is lower compared to other parasitoids in our study such as *Aphidius uzbekistanicus* (~500 eggs), *Aphidius matricariae* (~300 eggs) and *Praon volucre* (~350–500 eggs; Hagvar & Hofsvang, 1991; Lins, Bueno, Silva, Sampaio, & van Lenteren, 2011), whereas the longevities of the “tolerant” parasitoids *E. plagiator* (~15–25 days), *A. rhopalosiphi* (~13 days) and *A. ervi* (~15 days; Azzouz, Giordanengo, Wackers, & Kaiser, 2004; Malina & Praslicka, 2008) are longer compared to other parasitoids such as *A. matricariae* (~7–13 days) and *P. volucre* (~11 days; Hagvar & Hofsvang, 1991; Lins et al., 2011). This suggests that parasitoids with higher fecundity and lower longevity are more likely to oviposit unselectively, whereas species with a lower fecundity and higher longevity are more likely to overcome endosymbiont protection or to oviposit selectively.

In conclusion, our study on facultative endosymbiont–parasitoid occurrences in natural populations of *S. avenae* suggests that *H. defensa* and *R. insecticola* are widespread in this aphid species and that facultative endosymbionts confer considerable protection against a suite of parasitoid species. The primary defensive mechanism seems to be the killing of the immature parasitoid larva whereas a decrease in attractiveness of the aphid host to female primary parasitoids is a less important mechanism. The protection seems to be species‐specific for both endosymbionts and parasitoids, and it appears stronger when plants are fertilized. Our study also provides evidence that effects of facultative endosymbionts can cascade to higher trophic levels such as hyperparasitoids. At last, our analyses suggest that the two facultative endosymbiont species affect parasitoid communities and interactions independent from other environmental variables and as such can contribute to the reorganisation of interaction networks.

## AUTHORS’ CONTRIBUTIONS

Z.Y., I.V. and M.T. conceived and designed research; Z.Y., I.V. and N.P. conducted field experiment; Z.Y. and N.P. conducted the molecular work; Z.Y. and O.R. analysed the data; Z.Y. and M.T. wrote the manuscript. All authors read and contributed to the final version of the manuscript.

## DATA ACCESSIBILITY

The data of field experiment and molecular analysis have been archived on Dryad Digital Repository: https://doi.org/10.5061/dryad.s3b66h9 (Ye, Vollhardt, Parth, Rubbmark, & Traugott, 2018).
