# Supplementary material for: Facultative bacterial endosymbionts shape parasitoid food webs in natural host populations: A correlative analysis
Source: J Anim Ecol. 2018 Jul 16;87(5):1440–51. doi: 10.1111/1365-2656.12875 (PMC6099228; doi:10.1111/1365-2656.12875)
Supplement: Supplementary file 2 [file JANE-87-1440-s002.docx]

**Table S2** Number of randomly selected PCR products per target taxon which were amplified from field collected aphids and sequenced for confirming taxon identity. As no pea aphid X-type symbiont could be detected within the field samples, it was omitted from this list.

| **Target taxon** | **Number of sequenced amplicons** |
| --- | --- |
| *Sitobion avenae* | 4 |
| Aphidiinae | 2 |
| *Aphelinus*-Hyperparasitoid | 2 |
| *Dendrocerus* spp. | 2 |
| *Hamiltonella defensa* | 34 |
| *Regiella insecticola* | 34 |
| *Aphidius ervi* | 2 |
| *Aphidius avenae* | 2 |
| *Aphidius uzbekistanicus* | 2 |
| *Ephedrus plagiator* | 2 |
| *Praon gallicum* | 2 |
| *Praon volucre* | 2 |
| *Aphidius* spp. | 2 |
| *Aphelinus abdominalis* | 2 |
| *Alloxysta victrix* | 2 |
| *Asaphes suspensus* | 2 |
| *Asaphes vulgaris* | 2 |
| *Coruna clavata* | 1 |
| *Dendrocerus carpenteri* | 2 |
| *Phaenoglyphis villosa* | 2 |
