# Supplementary material for: Facultative bacterial endosymbionts shape parasitoid food webs in natural host populations: A correlative analysis
Source: J Anim Ecol. 2018 Jul 16;87(5):1440–51. doi: 10.1111/1365-2656.12875 (PMC6099228; doi:10.1111/1365-2656.12875)
Supplement: Supplementary file 3 [file JANE-87-1440-s003.docx]

**Table S3** Model fitting details of linear based analyses in this study.

| **Response** | **Parameters** |
| --- | --- |
| endosymbiont infection | aphid parasitism type* + plant fertilization + aphid parasitism type* × plant fertilization |
| primary parasitism | endosymbiont infection type** + plant fertilization + sampling date |
| hyperparasitism | endosymbiont infection type** + sampling date + endosymbiont infection type** × sampling date |

* Aphid parasitism type included three values: living unparasitized aphids, living parasitized aphids, and mummies.

** Endosymbiont infection included three values: uninfected, *Hamiltonella defensa*-infected, and *Regiella insecticola*-infected.
